# Supplementary material for: Scaffolds for Cartilage Tissue Engineering from a Blend of Polyethersulfone and Polyurethane Polymers
Source: Molecules. 2023 Apr 3;28(7):3195. doi: 10.3390/molecules28073195 (PMC10095814; doi:10.3390/molecules28073195)
Supplement: Supplementary file 1 [file molecules-28-03195-s001.zip › molecules-2256635-supplementary.pdf]

Scaffolds for Cartilage Tissue Engineering from a Blend of Polyethersulfone and Polyurethane Polymers

Monika Wasyłeczko [1,*], Elżbieta Remiszewska [1], Wioleta Sikorska [1], Judyta Dulnik [2] and Andrzej Chwojnowski [1]

[1] Nalecz Institute of Biocybernetics and Biomedical Engineering, Polish Academy of Sciences, Trojdena 4, 02-109 Warsaw, Poland; achwojnowski@ibib.waw.pl (A.C.)
[2] Institute of Fundamental Technological Research Polish Academy of Sciences, Laboratory of Polymers and Biomaterials, Pawińskiego 5b, 02-106 Warsaw, Poland
* Correspondence: mwasyleczko@ibib.waw.pl

1. Table S1 of Ion concentrations of degradation fluids.

Table S1. Ion concentrations of Hank's balanced salt solution (HBSS), simulated body fluid (SBF), and human blood plasma according to ISO 23317 [1,2].

**Ion**	**Contrecation (10^{-3} mol/L)**		
	**HBSS**	**SBF**	**Blood Plasma**
Na^+	141.60	142.0	142.0
K^+	5.81	5.0	5.0
Mg^{2+}	0.81	1.5	1.5
Ca^{2+}	1.26	2.5	2.5
Cl^-	144.80	147.8	103.0
HCO_3^-	4.09	4.2	27.0
HPO_4^{2-}	0.78	1.0	1.0
SO_4^{2-}	0.81	0.5	0.5

2. Table S2. The SEM photomicrographs of membranes after 2 and 4 weeks of degradation. For ease of description, the abbreviations used were MxYy, where x denoted the membrane number, Y- the degradation fluid (where Y for SBF was B, HBSS - H, and NaOH - N), and y denoted the number of the week. For example, M1B2 indicates the degradation of the membrane M1 in SBF after two weeks.

Table S2. The SEM photomicrographs of membranes after 2 and 4 weeks of degradation in SBF, HBSS, and 1M NaOH fluids. Scale bars: 300 μm – M1B2. M2B2, M2H2, M2N2, M2B4, M2H4, M2N4, M3N2, M3B4a, M3H4a, M3N4a, M4B2, M4H2, M4N2, M4H4a, M4N4a, M5B2, M5H2, M5H4a, M5N4a; 1000 μm – M1H2, M1N2, M1B4, M1H4, M1N4, M3B2, M3H2, M3H4b, M3N4b, M4B4a, M4B4b, M4H4b, M1N4b, M5N2, M5B4a, M5H4b, M5N4b; 2000 μm – M3B4b, M5B4b.

<table>
<tr><td>SBF</td><td>HBSS</td><td>1M NaOH</td></tr>
<tr><td colspan="3">M1 after 2 weeks of degradation</td></tr>
<tr><td>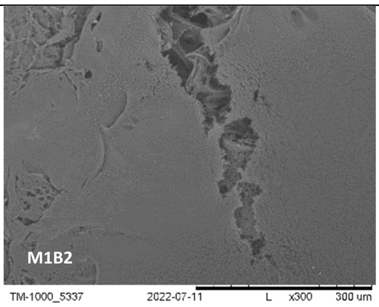
</td><td>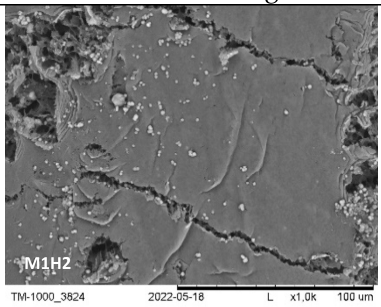
</td><td>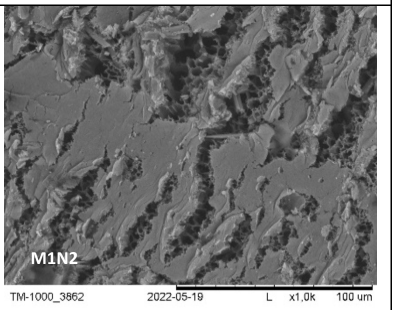
</td></tr>
<tr><td colspan="3">M1 after 4 weeks of degradation</td></tr>
<tr><td>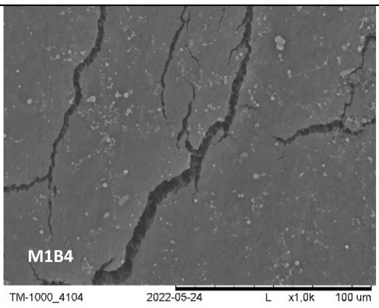
</td><td>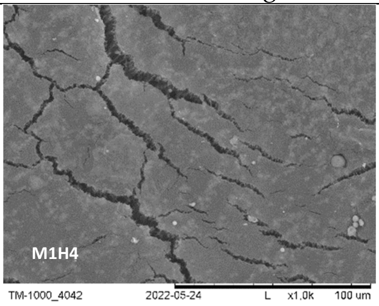
</td><td>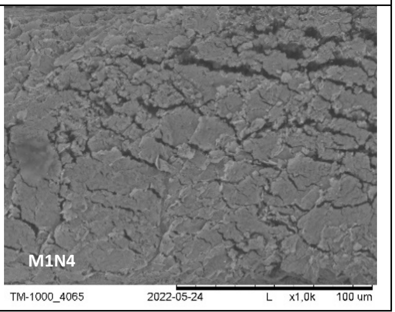
</td></tr>
</table>

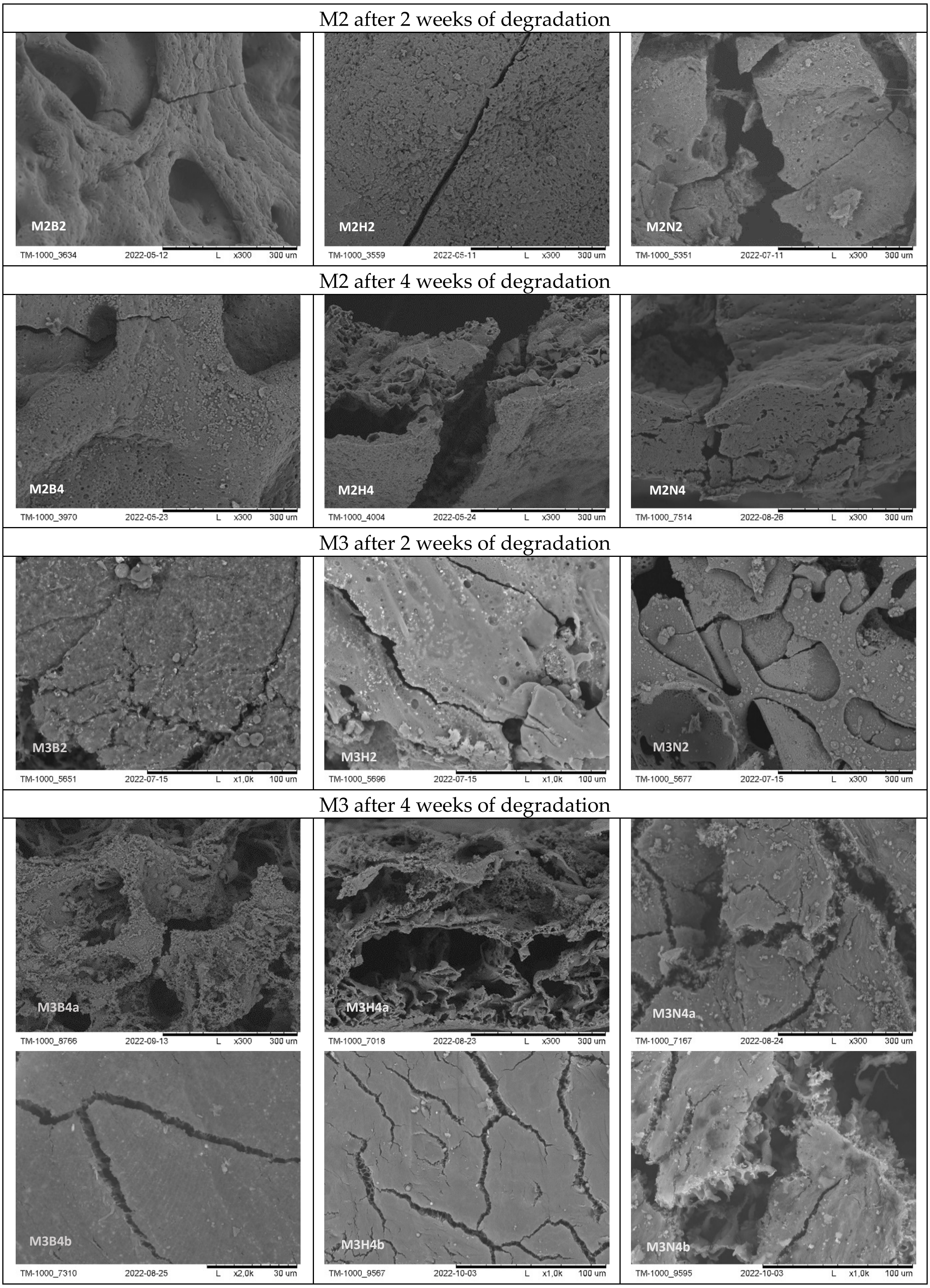
M2 after 2 weeks of degradation
M2B2
TM-1000_3634 2022-05-12 L x300 300 um
M2H2
TM-1000_3559 L x300 300 um
M2N2
TM-1000_5351 2022-07-11 L x300 300 um
M2 after 4 weeks of degradation
M2B4
TM-1000_3970 2022-05-23 L x300 300 um
M2H4
TM-1000_4004 2022-05-24 L x300 300 um
M2N4
TM-1000_7514 2022-08-26 L x300 300 um
M3 after 2 weeks of degradation
M3B2
TM-1000_5651 2022-07-15 L x1,0k 100 um
M3H2
TM-1000_5696 2022-07-15 L x1,0k 100 um
M3N2
TM-1000_5677 2022-07-15 L x300 300 um
M3 after 4 weeks of degradation
M3B4a
TM-1000_8766 2022-09-13 L x300 300 um
M3H4a
TM-1000_7018 2022-08-23 L x300 300 um
M3N4a
TM-1000_7167 2022-08-24 L x300 300 um
M3B4b
TM-1000_7310 2022-08-25 L x2,0k 30 um
M3H4b
TM-1000_9567 2022-10-03 L x1,0k 100 um
M3N4b
TM-1000_9595 2022-10-03 L x1,0k 100 um

M4 after 2 weeks of degradation

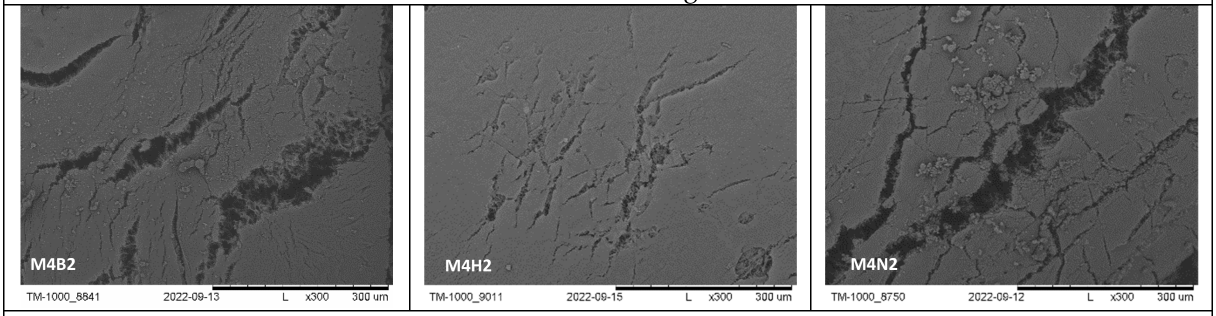

M4 after 4 weeks of degradation

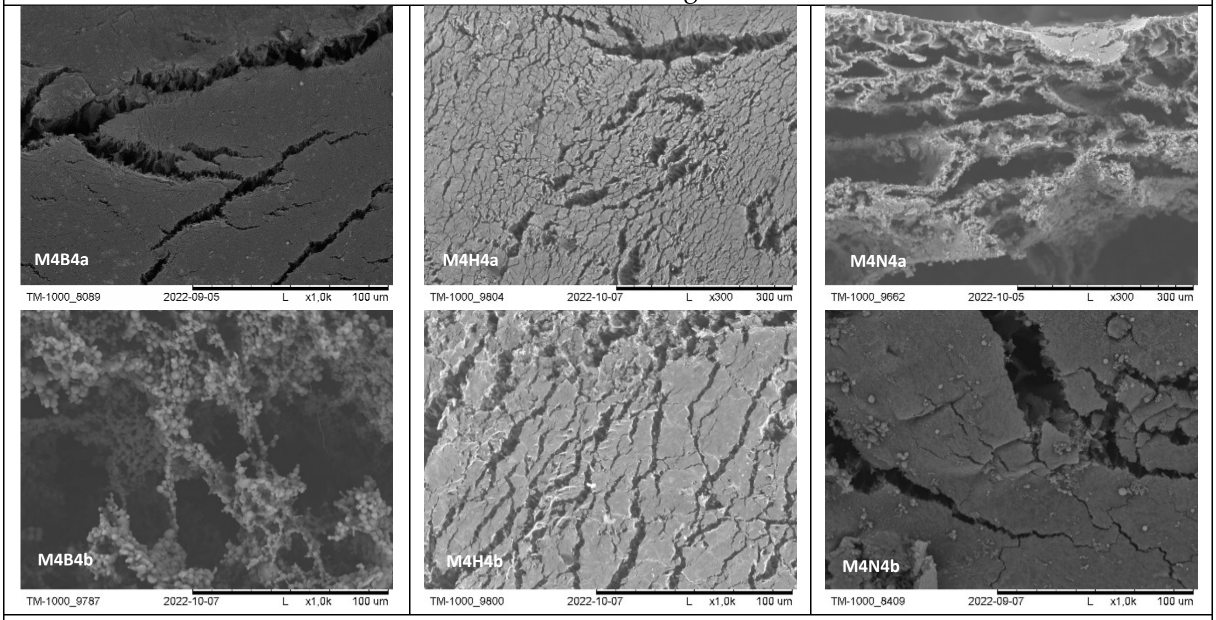

M5 after 2 weeks of degradation

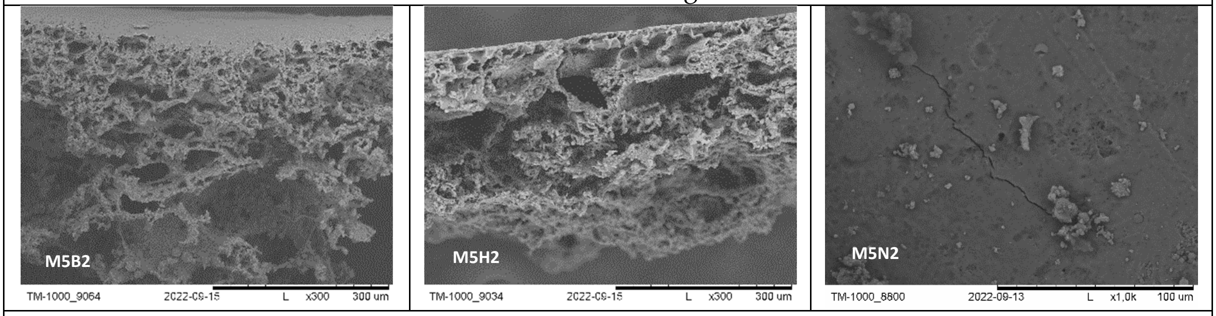

M5 after 4 weeks of degradation

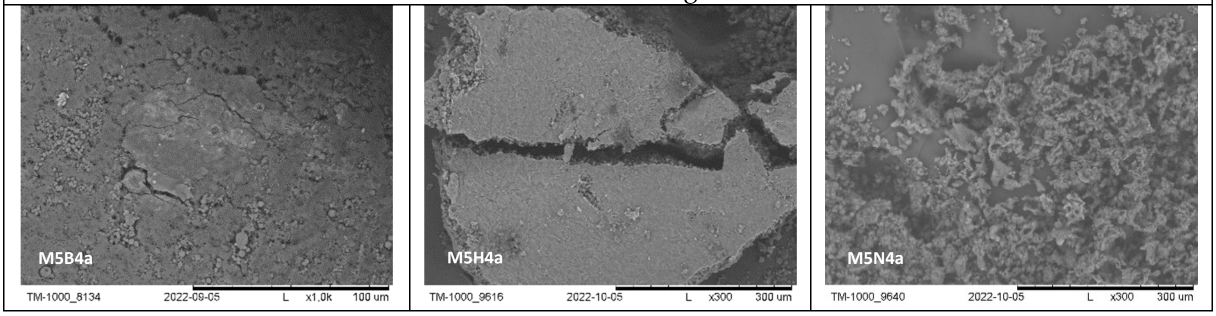

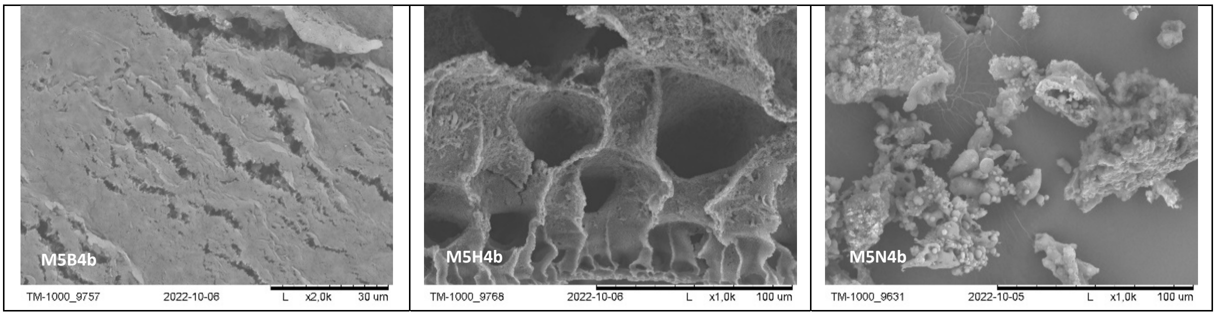

3. Figure S1. IR spectrum of M2-M5 membranes before, during and after degradation.

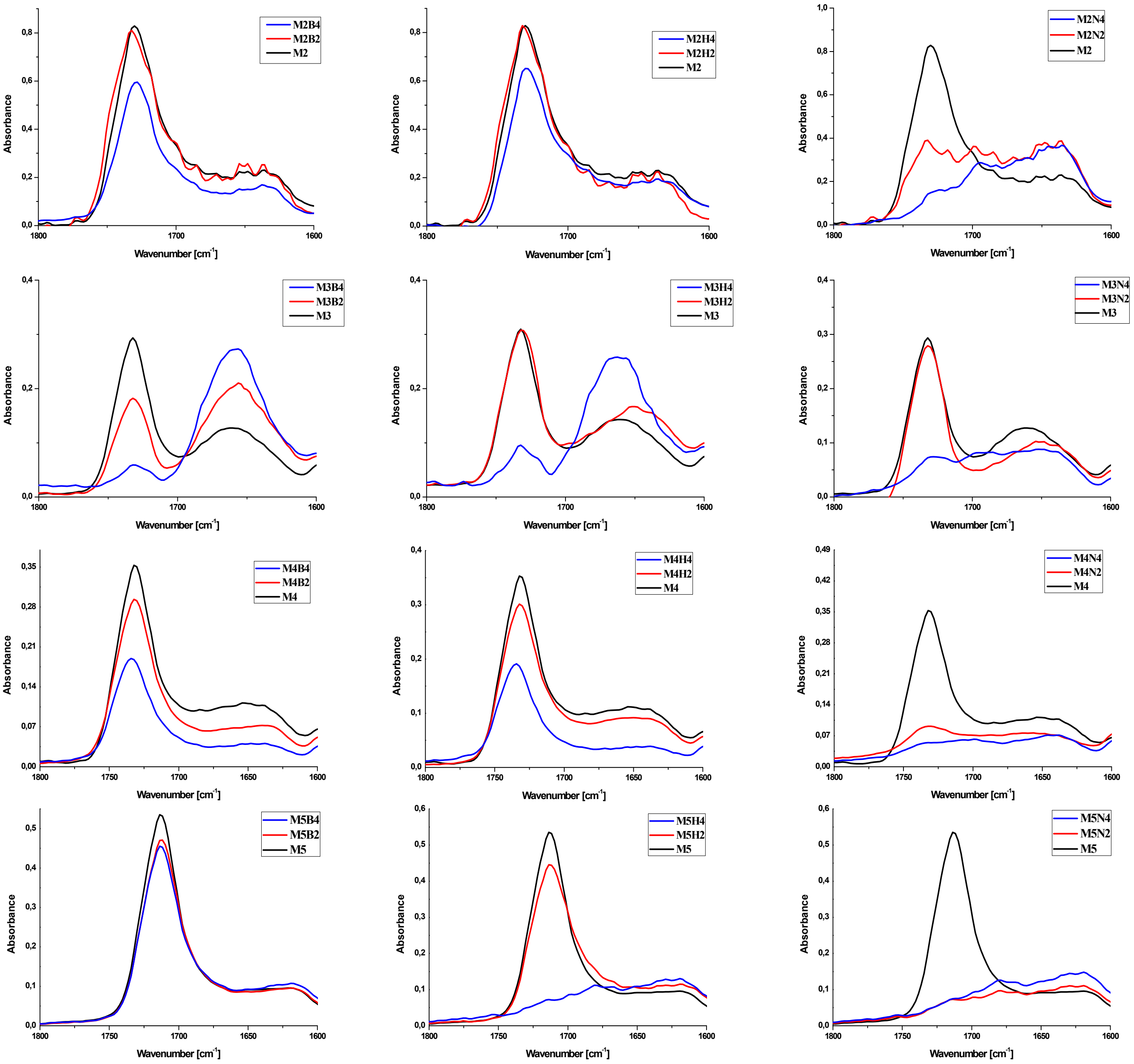

Figure S1. IR spectrum of M2-M5 membranes before and after 2 and 4 weeks of degradation in SBF (B), HBSS (H), and NaOH (N) fluid.

4. References

1. Chrzanowski, W.; Ali, E.; Neel, A.; Andrew, D.; Campbell, J. Effect of surface treatment on the bioactivity of nickel – titanium. *Acta Biomaterialia* **2008**, *4*, 1969–1984, doi:10.1016/j.actbio.2008.05.010.
2. Kokubo, T.; Takadama, H. How useful is SBF in predicting in vivo bone bioactivity? *Biomaterials* **2006**, *27*, 2907–2915, doi:10.1016/j.biomaterials.2006.01.017.
